# Chloroanisoles and Other Chlorinated Compounds in Cork from Different Geographical Areas

**DOI:** 10.3390/toxics7040049

**Published:** 2019-09-20

**Authors:** Pau Salvatella, Chantal Prat, Jordi Roselló, Enriqueta Anticó

**Affiliations:** 1 Francisco Oller S.A., 17244 Cassà de la Selva, Spain; pausalvatellasureda@gmail.com (P.S.); cprat@ollerfco.com (C.P.); jrosello@ollerfco.com (J.R.); 2 Department of Chemistry, University of Girona, 17003 Girona, Spain

**Keywords:** cork, chloroanisoles, SPE, geographical origin, *Armillaria mellea*

## Abstract

Cork quality is crucial for the fabrication of corks intended to be used to seal wine bottles. This work has focused on the determination of chloroanisoles (CAs)—exogenous compounds with a low perception threshold—in cork. The identification and quantification of these compounds was carried out with Bond Elut-ENV solid phase extraction and gas chromatography with mass spectrometry detection. Cork samples were obtained from oaks from Catalonia, Extremadura and Italy, and the presence of CAs was evaluated. Moreover, cork affected by the presence of yellow stains (a defect present in cork, mainly originated from the growth of the fungus *Armillaria mellea*) was analysed separately. The results obtained from cork macerates revealed the presence of trichloroanisole (TCA) in Catalan and Italian cork. Furthermore, TCA concentration was not statistically different when comparing cork affected and non-affected by the growth of *A. mellea*. Other chlorinated compounds were identified by comparison of their mass spectra with the data from the NIST library.

## 1. Introduction

Cork is a natural material obtained from the outer bark of *Quercus suber*. It is mainly used for the fabrication of cork stoppers employed in the wine industry for bottling. The main chemical compounds of cork include lignin (~25%, *w*/*w*), suberin (~40%, *w*/*w*) and polysaccharides (~20%, *w*/*w*) [1]. These compounds, forming a polymeric structure, accumulate in the cell wall of phellem cells of cork forming a barrier that prevents the permeation of water and gases among others. Apart from these more abundant components, cork contains small molecules (usually termed as extractives) and minerals that can migrate to water solution or wine. Extractives include aliphatic, triterpenic and phenolic molecules that contribute to the colour, flavour, astringency and bitterness of wines [2]. A special mention is devoted to the presence of chloroanisoles (CAs), bearing a very low perception threshold. Trichloroanisole (TCA) gives cork and wine a pronounced musty/mouldy odour, this defect being the origin of huge economical loses in the wine industry [3].

The use of chlorinated biocides in the forest or the washing procedures employing chlorine formerly applied in the cork industry have been described as the most probable sources of chloroanisoles and their precursors, chlorophenols. Álvarez-Rodríguez et al. reported the role of filamentous fungi in the O-methylation of chlorophenols [4]. Moreover, cork can be attacked by fungi such as *Armillaria mellea*, growing up into the bark of the tree, producing yellow stains and causing its death. *A. mellea* causes chemical and physical changes in cork, being responsible for the production of off-flavours [5].

Furthermore, it has been shown that the geographical origin of cork has an important role in its chemical properties. Conde et al. described the variability of the polyphenolic composition of Spanish cork from different provenances [6]. Jové et al. found significant differences for suberin and holocellulose with respect to the bark layer when cork from different production areas was studied [7].

The control of the chlorinated compounds, in particular CAs, is performed with gas chromatography (GC) using a selective detector such as the electron capture detector (ECD) or mass spectrometry (MS) [8]. The separation of the molecules from the matrix is a very important issue and several approaches have been developed. The soaking of the cork sample in a wine simulant, white wine, or water is used for the determination of the releasable fraction. Once the chlorinated compounds have been released in the aqueous matrix a preconcentration step is usually performed by solid phase extraction (SPE) or solid-phase microextraction (SPME) [3,9]. On the contrary, if the total concentration is sought, then an exhaustive solid–liquid extraction of cork is necessary, using apolar solvents like hexane. These methods were recently revised by Tarasov et al. [10].

In the present study, we developed a SPE–GC–MS method for the determination of CAs in cork samples. C18 cartridges were used and different experimental conditions (volume of sample, flow rate, elution solvent) were tested to achieve the detection limit for the quantification of the compounds in samples from different regions (Catalonia and Extremadura in Spain, and Italy). Moreover, cork affected by *A. mellea* and cork obtained from the outer part of the bark were also analysed.

## 2. Materials and Methods 

### 2.1. Chemicals and Reagents 

Separate stock solutions of CAs were purchased from Institut Català del Suro, Palafrugell, Spain. The concentration of the compounds was 100 µg·L^−1^, when prepared in methanol, and 100 mg·L^−1^, when prepared in hexane. In Table 1, the chemical structures and some properties of the CAs studied are presented.

n-Hexane (Panreac, Barcelona, Spain), dichloromethane (Romil, Leicestershire, United Kingdom) and absolute ethanol (Honeywell, Barcelona, Spain) were pesticide residue grade.

Calibration standards were prepared by diluting stock solutions in hexane in the range of 5 to 35 µg·L^−1^. These solutions were directly injected into the GC–MS and the equations for the calibration curves were *y* = 950*x* + 1675 (R^2^ = 0.998) for TCA, *y* = 1350*x* + 1644 (R^2^ = 0.999) for tetrachloroanisole (TeCA) and *y* = 566*x* + 1126 (R^2^ = 0.997) for pentachloroanisole (PCA). The limits of detection (LOD_cal_) were 1.1, 0.4 and 1.5 µg·L^−1^ for TCA, TeCA and PCA, respectively, calculated for a signal-to-noise ratio of three.

### 2.2. Cork Samples and Extraction

Cork bark samples (Table 2) were kindly supplied by Francisco Oller S.A., an important manufacturer of cork stoppers in Spain. In each bark, parts with yellow stains were separated. The outer surface of the bark was removed in some samples to compare its composition with the inner cork.

For the preparation of the clean cork macerates, i.e., without CAs, cork discs were used. The absence of CAs in the discs was previously checked.

Cork barks were milled to a ≤0.75 mm size with a ZM200 grinder (Retsch, Haan, Germany). The detachable parts of the grinder were cleaned between samples with compressed air.

Forty grams of the sample were carefully weighed and placed in a 1000 mL glass bottle. Then, they were extracted by adding 700 mL of hydroalcoholic solution ethanol/water (12% *v*/*v* ethanol) and left in an oven at 40 °C for 72 h. An ethanol/water mixture was chosen as the extractive solution because of its similarity to wine. Before the preconcentration and purification by applying SPE, the macerate was gravity and vacuum filtered in order to eliminate the suspended particles of cork.

The spiked macerates for the SPE evaluation were prepared by adding the appropriate volume of methanolic solution of CAs.

### 2.3. SPE Procedure

Bond Elut-ENV SPE cartridges (polystyrene divinylbenzene polymer, 500 mg, Agilent) were selected. The conditioning was carried out by applying 3 mL of methanol followed by 3 mL of 12% (*v*/*v*) ethanol/water. Ethanol/water 12% (*v*/*v*) solutions containing known amounts of CAs were used for the evaluation of the SPE procedure. The final SPE conditions were as follows: the sample was loaded at a flow-rate of 20 mL min^−1^; afterwards, the sorbent was dried under vacuum for 6 min; finally, elution of the analytes was carried out with 4 mL of hexane.

The eluate was concentrated with a gentle stream of nitrogen gas to a volume of 0.5 mL prior to gas chromatography analysis.

Results obtained in the SPE evaluation were compared in terms of recovery, *R* (%), calculated according to the following equation:

(1) $$R\left(\%\right)=\frac{C_{f}\times V_{f}}{C_{o}\times V_{o}}\times 100,$$

where *C_o_* represents the spiked concentration of the hydroalcoholic solution or the cork macerate, and *C_f_* is the concentration obtained from the peak area and using the calibration curves shown previously (Section 2.1). *V_o_* and *V_f_* are the volumes for the sample introduced in the SPE cartridges and the final volume (0.5 mL) before the chromatographic analysis, respectively.

### 2.4. Gas Chromatographic Conditions

Gas chromatography analysis was performed with a Trace GC Ultra equipped with a Polaris Q ion trap mass spectrometer (Thermo Scientific, Waltham, MA, USA) operating in the electron ionization mode at 70 eV. Splitless mode injections (1 µL) using a Triplus AS autosampler (Thermo Scientific, Waltham, MA, USA) were performed with the split valve opened at 1 minute. A BPX-5 capillary column (SGE) (30 m × 0.25 mm i.d., film thickness 0.25 µm) was used and helium was the carrier gas at 1 mL·min^−1^. The operating conditions were: an injector temperature of 270 °C; the oven temperature was programmed at 50 °C for 2 min, then increasing by 25 °C/min up to 110 °C and held for half a minute, afterwards the temperature was increased again by 2.5 °C/min up to 170 °C, held for 2 min and finally, increasing by 30 °C/min until 260 °C and held for 1 min. The ion source was set at 225 °C and the transfer line was held at 280 °C. Electron ionization mass spectra were recorded in the range *m*/*z* 40–300 amu (full scan mode). The chromatographic data were analysed by Xcalibur 1.4 software (Thermo Scientific, Waltham, MA, USA). NIST MS Search 2.0 library was used for the identification of compounds.

The following ions were selected for quantitative purposes: *m*/*z* = 210(75), 212(74) for TCA, corresponding to the M and M + 2 isotopes; *m*/*z* = 229(81), 231(100), 246(84) for TeCA, where 229 and 231 correspond to loss of the methyl group from M and M + 2 isotopes, and 246 correspond to the M + 2 isotope of TeCA; and *m*/*z* = 265(99), 267(65) for PCA, corresponding to loss of the methyl group from M + 2 and M + 4 isotopes. The theoretical relative intensity of the fragments is given in parenthesis.

## 3. Results and Discussion

### 3.1. SPE Method Development

C18 and polymeric sorbents have been successfully used in other investigations for the study of CAs in wines [3,12]. Usually in SPE, after sorbent conditioning and sample loading, an intermediate washing step is implemented to eliminate some of the interferences, before the elution of the compounds of interest. The washing can be a critical stage since only the interfering compounds should be rinsed through with the washing solutions while leaving the compounds of interest behind. In this study, we have assayed the direct elution of the analytes without a washing step that may cause losses of the compounds of interest. Then, SPE was evaluated by testing different drying times, flow rates, and sample volumes. As for the elution solvent, the use of hexane and dichloromethane was considered, since both organic solvents have previously been used for the solubilisation of CAs from cork and other matrices [13].

The recovery results for the different drying times tested are shown in Table 3. Fortified hydroalcoholic solution (100 mL) with a concentration of 0.15 µg·L^−1^ for each compound was used, and dichloromethane and hexane were both tested for the elution.

Considering the results obtained for the two replicates, similar recoveries were observed for the dichloromethane elution at different drying times. In the case of hexane, 1 and 6 min were compared and we found that for 1 min drying time, the recoveries of the three CAs were much lower (8%, 19% and 44% for TCA, TeCA and PCA, respectively). The different behaviour observed between the two solvents was probably due to there being water residues present in the sorbent when 1 min drying time was applied. Hexane is a non-polar solvent with a lower tendency to mix with the remaining water, and therefore a longer drying time should be applied. According to these results, a drying time of 6 min was selected for further experiments.

Next, the influence of flow rate was tested at 10 and 20 mL·min^−1^. No difference in the recoveries was found between the flow rates. Therefore, the higher one was selected as the most convenient in terms of sample throughput.

**Figure 1:**
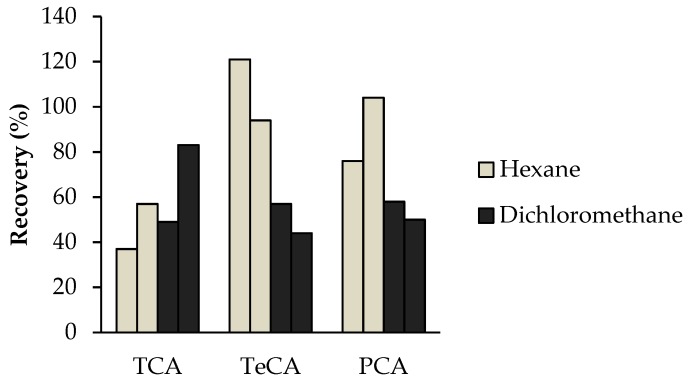
Comparison of the recoveries obtained for each solvent, using 100 mL of fortified hydroalcoholic solution, 6 min drying time and 20 mL min^−1^ flow rate (*n* = 2).

Using the conditions previously mentioned, the results obtained for hexane and dichloromethane were compared in Figure 1. Better results were achieved with hexane for TeCA and PCA. However, recoveries for TCA were similar for both solvents. Taking into account these results, both hexane and dichloromethane were used in the evaluation of the matrix effect.

### 3.2. Matrix Effect and Quality Parameters of the Method

The matrix effect has been studied by comparing the recoveries of the target compounds when using a spiked cork macerate. These macerates usually contain a higher amount of extractives like sugars, phenolic compounds, terpenes and terpenoids, among others. These molecules can also interact with the C18 sorbent of the SPE cartridge, decreasing the available sites for the retention of the CAs. This would negatively affect the recoveries of the compounds and increase the method detection limits. For that, we evaluated the matrix effect by comparing 100 and 500 mL of spiked cork macerate using hexane or dichloromethane as eluents. The spiking level was 0.15 µg·L^−1^ for the 100 mL sample and 0.03 µg·L^−1^ for the 500 mL sample. The results are collected in Table 4.

As can be seen, recoveries were largely decreased when the sample volume increased. Nevertheless, in order to obtain the required detection and quantification limits (Table 5), the 500 mL samples were used in the following studies. It is worth mentioning that the work of Soleas et al. was the only one using SPE for the quantification of CAs in cork. They claimed that the recoveries were good, however they used macerates obtained from whole cork stoppers and the contact time was shorter (48 h) [3]. These mild conditions made the matrix simpler than in our case.

Taking into account the results in Table 4, the limit of detection (LOD) of the method was calculated, by using the following equation:

(2) $$LOD\left(\frac{ng}{L}\right)=\frac{LOD_{cal}\left(\frac{ng}{L}\right)\times 0.5\cdot 10^{-3}L}{0.5L}\times \frac{100}{R},$$

where *LOD_cal_* are the values reported in Section 2.1, and *R* are the recovery values obtained from Table 4, using hexane for the elution and a 500 mL sample volume.

In Table 5 LODs are shown for the three CAs. The limit of quantification (LO) was calculated in a similar manner as LODs but for a signal-to-noise ratio equal to 10.

It is worth mentioning that the values above are the same order of magnitude as the values found in the work of Martínez-Uruñuela et al. [12] for wines. However, they are higher than the LOD reported for TCA in Soleas et al. [3]. We believe that an appropriate washing step would improve the recovery values and in turn decrease the LOD of the method.

### 3.3. Results for the Samples of Different Geographical Origins

**Figure 2:**
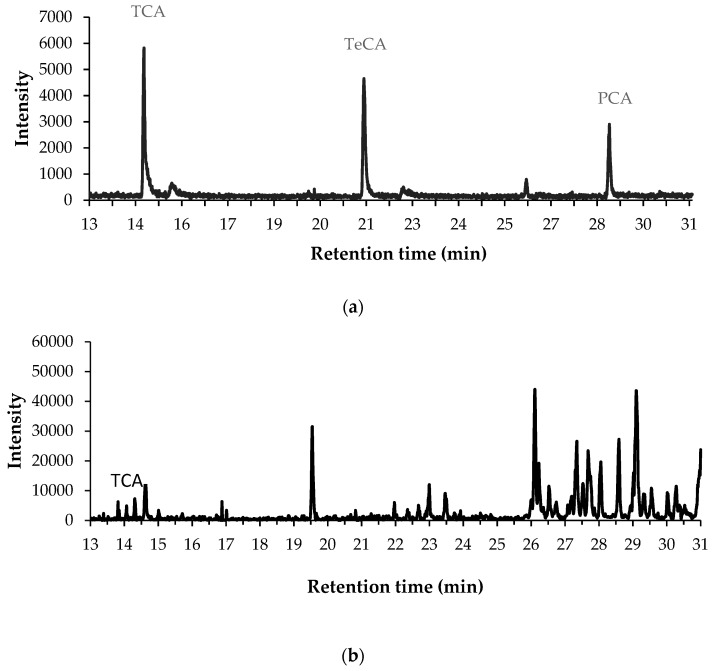
(**a**) Extracted chromatogram (*m*/*z* 210,212,229,231,246,265,267) for a standard solution (35 µg·L^−1^) prepared in hexane and directly injected into the GC; (**b**) extracted chromatogram (*m*/*z* 210,212) for C1N cork macerate after applying the SPE method.

Using the previous method, the macerates obtained from corks of different origin were examined. In Figure 2, a chromatogram for the sample C1N together with the chromatogram for a standard in hexane are depicted. The presence of TCA in the cork sample was evidenced from the coincidence of the retention time, and also from the isotope ratios in both spectra.

In Table 6, the concentrations of chloroanisoles found in samples without yellow stains are shown. The calculation of the total concentration in the cork samples was performed using Equation (2) and substituting the LOD_cal_ for the concentration obtained in the chromatographic analysis of the hexane solution obtained after elution of the cartridge.

As can be observed, TCA was the only chloroanisole found in samples from Catalonia and Italy. The cork samples from the three regions were free from TeCA and PCA. These results agree with the sensory data reported from a sensory panel in the cork producer, being that the cork purchased from Extremadura was the one that presented with a lower incidence of defects. These findings open a window for new investigations that could be of paramount importance for ensuring the quality of cork stoppers produced in the cork industry.

**Figure 3:**
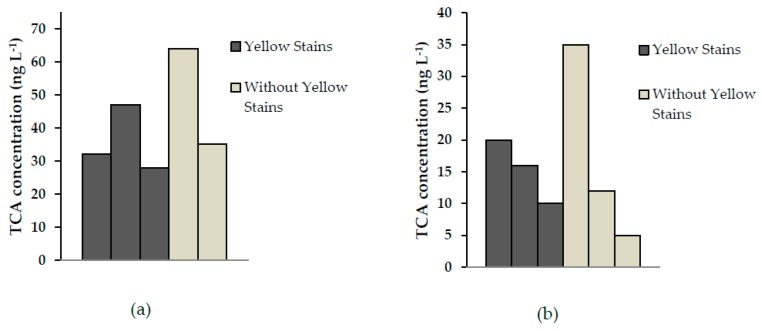
Comparison of the trichloroanisole (TCA) concentration in samples from (**a**) Catalonia, and (**b**) Italy with and without yellow stains.

A comparison of TCA between cork samples with and without yellow stains is shown in Figure 3.

According to the results shown in Figure 3, no differences in TCA concentration were observed in cork bark not affected by *A. mellea* and the corresponding cork presenting yellow stains. These results indicated that the presence of TCA in cork probably had different origins apart from the microbial activity of *A. mellea*. In the study of Rocha et al. [5], the authors conclude that cork affected by *A. mellea* presented larger amounts of lignin-related derivatives. The possibility of their eventual chlorination and transformation into CAs has currently diminished considerably because the use of chlorine is avoided in the production of cork stoppers.

Finally, we investigated the presence of TCA in samples I3NN, C3NN and S3NN where the outer part of the bark was removed. We found TCA concentration below the limit of quantification of the method. Accordingly, it seems that the presence of CAs could be closely related to the presence of these compound in the environment surrounding the forests, followed by the adsorption in the cork bark. This hypothesis needs to be further investigated in the future.

### 3.4. Identification of Other Chlorinated Compounds

Some other chlorinated compounds were identified from the chromatographic data, by comparing their mass spectra with the NIST library. These are represented in Table 7 together with their probability of identification.

The identified compounds belong to the group of chlorinated lignin derivatives, and they were all found in samples from the Catalonia region. The presence of these compounds should be confirmed by the analysis of pure standards.

## 4. Conclusions

In this work, we evaluated the extraction of chloroanisoles from ground cork samples from different geographical origins with hydroalcoholic solutions as extractant combined with SPE preconcentration and GC–MS analysis. The determined SPE conditions were: 500 mL cork macerate applied to the C18 cartridge at a flow rate of 20 mL min^−1^, 6 min drying time, and elution with 4 mL hexane. Using these conditions, the LOQ for TCA, TeCA and PCA were in the low ng·g^−1^ range. From our results, we found that the only CA present was TCA in samples from Catalonia and Italy, while samples from Extremadura were found to be free of CAs contamination. *A. mellea* seemed not to be related to the presence of CAs. Both, the influence of the geographical origin and the participation of *A. mellea* in the formation of CAs need to be further investigated.

## Figures and Tables

**Table 1:** Structures, molecular weights and perception threshold of chloroanisoles in wines.

Structure	Name	Average Molecular Weight	Perception Threshold in Wine and Sensory Attributes [11]
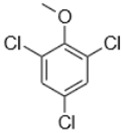	2,4,6-trichloroanisole (TCA)	211.47	3 ng·L^−1^ Musty, mouldy
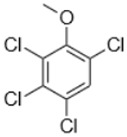	2,3,4,6-tetrachloroanisole (TeCA)	245.91	5–10 ng·L^−1^ Musty, rancid
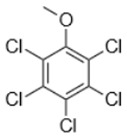	2,3,4,5,6-pentachloroanisole (PCA)	280.35	5 µg·L^−1^ Musty, rancid

**Table 2:** Origin and classification of each sample depending on whether they had yellow stains or not. In cork samples labelled as C3NN, E3NN and I3NN, the outer cork part was removed.

Cork Origin	Samples	Classification	Sample Code
Catalonia	Bark 1	Yellow Stains	C1Y
		No Yellow Stains	C1N
	Bark 2	Yellow Stains	C2Y
		No Yellow Stains	C2N
	Bark 3	Yellow Stains	C3Y
		No Yellow Stains No Yellow Stains or Outer Bark	C3N C3NN
Extremadura	Bark 1	Yellow Stains	E1Y
		No Yellow Stains	E1N
	Bark 2	Yellow Stains	E2Y
		No Yellow Stains	E2N
	Bark 3	Yellow Stains	E3Y
		No Yellow Stains No Yellow Stains or Outer Bark	E3N E3NN
Italy	Bark 1	Yellow Stains	I1Y
		No Yellow Stains	I1N
	Bark 2	Yellow Stains	I2Y
		No Yellow Stains	I2N
	Bark 3	Yellow Stains	I3Y
		No Yellow Stains No Yellow Stains or Outer Bark	I3N I3NN

**Table 3:** Recoveries obtained from 100 mL of fortified hydroalcoholic solutions, by eluting them with dichloromethane after a drying step of 1, 6 or 30 min.

	Recovery (%)					
	1 min		6 min		30 min	
	Replicate 1	Replicate 2	Replicate 1	Replicate 2	Replicate 1	Replicate 2
TCA	51	48	49	44	40	40
TeCA	52	49	83	58	48	60
PCA	60	56	57	50	51	48

**Table 4:** Recoveries for dichloromethane and hexane solvents, using fortified macerates of 100 and 500 mL. A flow rate of 20 mL·min^−1^ and a drying time of 6 min was used in all cases.

Recovery (%)							
	Dichloromethane			Hexane			
	100 mL		500 mL	100 mL		500 mL	
	Replicate 1	Replicate 2	Replicate 1	Replicate 1	Replicate 2	Replicate 1	Replicate 2
TCA	60	27	16	69	37	28	30
TeCA	78	51	22	109	75	27	29
PCA	56	31	26	85	89	39	41

**Table 5:** Limits of detection and quantification for the method described.

	LOD (ng·L^−1^)	LOD (ng·g^−1^)	LOQ (ng·L^−1^)	LOQ (ng·g^−1^)
TCA	4	0.07	10	0.2
TeCA	1	0.02	5	0.08
PCA	4	0.07	12	0.2

**Table 6:** Chloroanisoles found in cork samples without yellow stains from Catalonia (two replicates), Extremadura and Italy in ng·g^−1^.

		TCA	TeCA	PCA
**Catalonia**	Bark 1	0.9; 1.2	<LOD	<LOD
	Bark 2	0.4; 0.7	<LOD	<LOD
	Bark 3	-	-	-
**Extremadura**	Bark 1	<LOD	<LOD	<LOD
	Bark 2	<LOD	<LOD	<LOD
	Bark 3	<LOD	<LOD	<LOD
**Italy**	Bark 1	0.6	<LOD	<LOD
	Bark 2	0.2	<LOD	<LOD
	Bark 3	0.09	<LOD	<LOD

**Table 7:** Chlorinated compounds identified in cork samples, *m*/*z* of the three most abundant fragments with their theoretical relative intensity in brackets and the coincidence probability according to the NIST library.

Identified Compound	Positive Samples	Coincidence Probability	Most Abundant Fragments
1,2,4,5-tetrachloro-3,6-dimetoxybenzene			
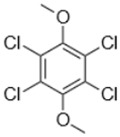	C1Y	92%	261(100), 259(79), 276(68)
	C1N C2Y C2N	87% 95% 70%	
1-chloro-2,3,4-trimethoxybenzene			
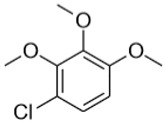	C1Y	63%	202(100), 187(47), 204(34)
	C1N C2Y	51% 22%	
